# Supplementary material for: Helicobacter pylori infection and eradication outcomes among Vietnamese patients in the same households: Findings from a non-randomized study
Source: PLoS One. 2021 Nov 24;16(11):e0260454. doi: 10.1371/journal.pone.0260454 (PMC8612553; doi:10.1371/journal.pone.0260454)
Supplement: S1 Table — (DOCX) [file pone.0260454.s001.docx]

**S1 Table. Characteristics of *H. pylori*-treated patients who did and did not return for follow-up within 4 months.**

| **Characteristics** | **Did not return (n=598)** | **Returned (n=341)** | **p-value**^a^ |
| --- | --- | --- | --- |
| **Gender** |  |  | 0.39 |
| Female | 323 (62.5) | 194 (37.5) |  |
| Male | 275 (65.2) | 147 (34.8) |  |
| **Age (years)** |  |  | 0.11 |
| <12 | 131 (57.5) | 97 (42.5) |  |
| 12 to 18 | 61 (64.2) | 34 (35.8) |  |
| >18 to <45 | 306 (66.9) | 151 (33.1) |  |
| ≥45 | 100 (62.9) | 59 (37.1) |  |
| **Family membership** |  |  | 0.36 |
| Father | 160 (67.2) | 78 (32.8) |  |
| Mother | 220 (64.3) | 122 (35.7) |  |
| Son | 115 (62.5) | 69 (37.5) |  |
| Daughter | 103 (58.9) | 72 (41.1) |  |
| **Regimen**^b^ |  |  | 0.009 |
| Clarithromycin sequential^c^ | 66 (51.2) | 63 (48.8) |  |
| Tetracycline sequential^d^ | 488 (65.7) | 255 (34.3) |  |
| Bismuth quad^e^ | 38 (63.3) | 22 (36.7) |  |

^a^Differences were tested by Chi-square test.

^b^Levofloxacin regimen was excluded due to small sample size (n=1).

^c^Clarithromycin sequential regimen: esomeprazole, amoxicillin, and bismuth for the first 7 days, esomeprazole, clarithromycin, metronidazole, and bismuth for the second 7 days.

^d^Tetracycline sequential regimen: esomeprazole, amoxicillin, and bismuth for the first 7 days, esomeprazole, metronidazole, tetracycline, and bismuth for the second 7 days.

^e^Bismuth quad regimen: esomeprazole, amoxicillin, metronidazole, and bismuth for 14 days.
