# Supplementary material for: Helicobacter pylori infection and eradication outcomes among Vietnamese patients in the same households: Findings from a non-randomized study
Source: PLoS One. 2021 Nov 24;16(11):e0260454. doi: 10.1371/journal.pone.0260454 (PMC8612553; doi:10.1371/journal.pone.0260454)
Supplement: Attachment — Submitted filename: Response to Reviewers.docx [file pone.0260454.s002.docx]

We would like to thank the reviewer for their insightful comments on our manuscript. Please find below our responses to the comments.

**1. The study is important and addresses an important research question regarding the antibiotic eradication of *H. pylori* among the familial contacts of *H. pylori* patients.**

Thank you.

**2. There are 4 *H. pylori* eradication regimens. How were patients distributed among the 4 regimens? Was any randomization done? Was the distribution blind? How was the number in each group arrived at? Any NNT done? Is the study powered enough to analyse the differences in *H. pylori* eradication rates of different regimens?**

This was not a randomized controlled trial. Since our primary outcome was the prevalence of *H. pylori* infection among people living in the same households, we conducted a cohort study and did not plan on randomizing the treatment regimens. Treatment was decided by the examining doctor (clarithromycin and tetracycline regimens were first-line regimens, and bismuth quad and levofloxacine regimens were second-line). Therefore, we did not calculate sample size and power for the secondary outcome (treatment outcome).

**3. We see that levofloxacin-based regimen was second line treatment but had only 1 patient. Does it mean there was only one *H. pylori* eradication failure? If not, what happened to others? What were the outcomes in them?**

We have discussed with the doctors who treated the study patients. Some also used the bismuth quad regimen for patients with history of failure to eradicate. Therefore, we have revised our manuscript to reflect this (see Lines 112-113).

**4. Non-adherence to antibiotic protocols. It would have easily found how much non-adherence to antibiotic protocol resulted in failure to treatment. Was anything done to improve follow up. What is experience of other articles/authors regarding this issue of follow up?**

Non-adherence is important but very difficult to resolve. Although *H. pylori* infection is common, it is not considered a serious condition in Vietnam, and many patients do not present with severe symptoms. Therefore, they may miss some doses, stop the treatment as soon as they feel better, and decide not to return for follow-up. As described in our study, only more than 1/3 of the patients who received treatment returned for follow-up.

In a clinical trial on *H. pylori* that we have just finished, we followed up study participants after 2 weeks since enrollment. A substantial portion of patients forgot some doses during the 2 weeks of therapy (we couldn't share the specific number since it's not published), and some did not agree to return for follow-up. Our strategy was to explain to them how adherence to *H. pylori* treatment is important (emphasizing the risk of antibiotic resistance), but the practice did not improve in many patients. We think a broader approach must be sought, for example, mass communication through television and online channels.

**5. Conclusions mentioned in the abstract and in the final paper do not match.**

We have revised both the conclusions in the abstract and full text.

**6. Follow up patients is very less ~33% (341/939). Authors have not offered any explanation for the same.**

We mentioned the significant loss-to-follow-up rate in the paragraph about limitations in the Discussion section. We have expanded the discussion on this issue in the paragraph (see Lines 276–279).

**The study main and important issue was familial transmission regarding H. pylori. Unfortunately, the conclusion says nothing about this point.**

To attribute the higher prevalence among people in the same households to familial transmission, we will need a longitudinal study where the entire households are sampled from the general populations and followed up over time. We deemed our findings inadequate to conclude anything on familial transmission; therefore, we only stated that the prevalence among people who live in the same households was high (and from our discussion, higher than previously reported numbers).
