# Supplementary material for: Helicobacter pylori infection and eradication outcomes among Vietnamese patients in the same households: Findings from a non-randomized study
Source: PLoS One. 2021 Nov 24;16(11):e0260454. doi: 10.1371/journal.pone.0260454 (PMC8612553; doi:10.1371/journal.pone.0260454)
Supplement: Attachment — Submitted filename: Response to Reviewers.docx [file pone.0260454.s003.docx]

We would like to thank the reviewer for their insightful comments on our manuscript. Please find below our responses to the comments.

**1. The title should be changed suitably to reflect that this is not a randomised study.**

We have added a few words to clariy that this is a non-randomized study.

**2. As the authors accepted that they have not calculated NNT and it may not be adequately powered study to study the differences between the different H. pylori treatment regimens, the same should be mentioned in the methods the manuscript may be accepted after incorporating the above changes.**

We have added a sentence to clarify that no sample size and power calculation was done for the treatment outcomes (see the Revised Manuscript, lines 114-116).
